# ggMOB: Elucidation of genomic conjugative features and associated cargo genes across bacterial genera using genus-genus mobilization networks

**DOI:** 10.3389/fgene.2022.1024577

**Published:** 2022-12-08

**Authors:** Gowri Nayar, Ignacio Terrizzano, Ed Seabolt, Akshay Agarwal, Christina Boucher, Jaime Ruiz, Ilya B. Slizovskiy, James H. Kaufman, Noelle R. Noyes

**Affiliations:** ^1^ Department of Biomedical Informatics, Stanford University, Stanford, CA, United States; ^2^ IBM Research Almaden, San Jose, CA, United States; ^3^ Department of Computer and Information Science and Engineering, University of Florida, Gainesville, FL, United States; ^4^ Department of Veterinary Population Medicine, University of Minnesota, Minneapolis, MN, United States; ^5^ Altos Labs, Redwood City, CA, United States

**Keywords:** antimicobial resistance, genomics, metagenomics, big data, mobile genetic elements (MGE)

## Abstract

Horizontal gene transfer mediated by conjugation is considered an important evolutionary mechanism of bacteria. It allows organisms to quickly evolve new phenotypic properties including antimicrobial resistance (AMR) and virulence. The frequency of conjugation-mediated cargo gene exchange has not yet been comprehensively studied within and between bacterial taxa. We developed a frequency-based network of genus-genus conjugation features and candidate cargo genes from whole-genome sequence data of over 180,000 bacterial genomes, representing 1,345 genera. Using our method, which we refer to as ggMOB, we revealed that over half of the bacterial genomes contained one or more known conjugation features that matched exactly to at least one other genome. Moreover, the proportion of genomes containing these conjugation features varied substantially by genus and conjugation feature. These results and the genus-level network structure can be viewed interactively in the ggMOB interface, which allows for user-defined filtering of conjugation features and candidate cargo genes. Using the network data, we observed that the ratio of AMR gene representation in conjugative *versus* non-conjugative genomes exceeded 5:1, confirming that conjugation is a critical force for AMR spread across genera. Finally, we demonstrated that clustering genomes by conjugation profile sometimes correlated well with classical phylogenetic structuring; but that in some cases the clustering was highly discordant, suggesting that the importance of the accessory genome in driving bacterial evolution may be highly variable across both time and taxonomy. These results can advance scientific understanding of bacterial evolution, and can be used as a starting point for probing genus-genus gene exchange within complex microbial communities that include unculturable bacteria. ggMOB is publicly available under the GNU licence at https://ruiz-hci-lab.github.io/ggMOB/

## 1 Introduction

Several mechanisms of horizontal gene transfer (HGT) allow bacteria to exchange genetic material. One of these mechanisms, termed conjugation, occurs when bacterial cells form direct physical contacts that allow for passage of genetic material from one bacterium to another. The machinery required to form these contacts and initiate genetic exchange is often contained within integrative and conjugative elements (ICE), plasmids, and other mobile genetic elements (MGEs) (Frost et al., 2005; Wozniak and Waldor, 2010; Roberts and Mullany, 2011; Wiedenbeck and Cohan, 2011; Perry and Wright, 2013; Johnson and Grossman, 2015; Singer et al., 2016). The conditions that induce excision and conjugation are not fully elucidated, but DNA damage and subsequent SOS response seem to be an important trigger (Waldor et al., 2004; Koraimann and Wagner, 2014). The cost of acquiring and maintaining the new genetic material also influences the success of transfer events (Uhlemann et al., 2021).

Genes exchanged between bacteria during conjugation include functional domains associated with conjugative machinery (e.g., excisionases, integrases, conjugative transport proteins) as well as intervening “accessory” genes that are not necessary for conjugation, often termed “cargo genes” (Johnson and Grossman, 2015). By pairing conjugative machinery with an array of diverse cargo genes, bacterial communities can significantly expand their genetic repertoire, including between bacteria of diverse taxonomy (Guglielmini et al., 2011; Bellanger et al., 2014; Neil and Allard, 2021). Functions commonly associated with conjugative cargo include antimicrobial resistance (AMR) and virulence (Roberts and Mullany, 2011; Perry and Wright, 2013; Johnson and Grossman, 2015; Cury et al., 2017), which can pose a risk to human and animal health if transferred into pathogens (Partridge et al., 2018). Therefore, understanding the microbial ecology of conjugative elements and cargo genes (i.e., their distribution and behavior across bacterial taxa) is important in assessing the risk posed by various bacterial communities (Gaston et al., 2021). For example, how often do different bacterial taxa carry conjugative machinery and AMR genes; what resistance phenotypes are commonly associated with the presence of conjugative machinery within the genome; how often do different commensal bacterial taxa carry out conjugation to exchange cargo genes with pathogens; and what conditions foster conjugative exchange of specific cargo genes between pathogens and non-pathogens? These questions are fundamental to understanding how bacterial communities respond to external stimuli, and how these responses increase the overall risk posed by microbial communities of varying composition (Martínez et al., 2015; Oh et al., 2018).

However, the process of conjugative HGT is highly stochastic and therefore, difficult to predict (Lopatkin and Collin, 2020). One reason for this stochasticity is variability in the conjugation competency of donor and recipient bacterial cells for a given conjugative MGE; as well as variability in the capacity of a given type of conjugative MGE to also transfer unrelated cargo genes. Recent meta-analyses of conjugation rates for specific bacterial species and/or MGEs have highlighted these complexities (Alderliesten et al., 2020; Sheppard et al., 2020). Historically, the scientific process for estimating conjugative likelihood has stemmed from highly controlled *in vitro* experiments between pairs of bacterial isolates and specific MGEs. Results from such studies have been crucial for uncovering the behavior of MGEs and their importance for functions such as AMR. However, reductive experiments typically do not generalize well to the complex microbial communities found *in situ*, including host and environmental microbiomes. Furthermore, these experiments are necessarily restricted in their ability to characterize the full bacterial host range of a given MGE, as they typically involve only several distinct bacterial taxa. One major challenge that remains is to generate a conjugation likelihood for every host-donor-MGE combination observed across all bacterial taxa and MGE.

Insight into this challenge can be gained through the plethora of whole genome sequence (WGS) data which is now publicly available. As an example, the analysis of HGT-associated genes from just 336 genomes across 16 phyla was sufficient to significantly improve bacterial phylogenies as compared to those obtained from conserved marker genes (Abby et al., 2012). An analysis of 1,000 genomes demonstrated that ICE machinery is ubiquitous across diverse prokaryotes, and likely one of the most common mechanisms of bacterial evolution (Guglielmini et al., 2011). Currently, public datasets contain orders of magnitude more WGS data, which can be used to improve our understanding of the mechanisms by which critically important genes and pathogens emerge and persist (Botelho et al., 2020). However, despite the importance of HGT in bacterial evolution and pathogenicity, there has not yet been a comprehensive, systematic survey of the frequency of conjugation and cargo genes within or between bacterial genera. The objective of this work was to describe intra- and inter-genus conjugation-cargo dynamics by leveraging the comprehensive set of WGS data and conjugation sequences currently available within the Reference Sequence (RefSeq) and Short Read Archive (SRA) databases at the National Center for Biotechnology Information (NCBI). In particular, we analyzed 186,887 WGS datasets to identify putative conjugation events and corresponding candidate cargo genes, as well as to characterize the frequency of AMR genes with respect to the frequency of their occurrence with conjugative proteins. We were able to identify over 95,000 genomes containing conjugative proteins, and more than 4 billion putative cargo genes between genomes. We summarize and disseminate this analysis through an open-source network that describes the genus-genus sharing of conjugation features and cargo genes, representing genomes from over 1,300 different genera. Our network, which we refer to as ggMOB, allows users to filter for both conjugation features and putative cargo genes. Using ggMOB we analyzed the ratio of AMR gene representation in conjugative *versus* non-conjugative genomes and found it to be greater than 5:1, confirming that conjugation is a critical force for AMR spread across genera. Finally, we demonstrated that clustering genomes by conjugation profile sometimes correlated well with taxonomic structuring, but in some cases was highly discordant, suggesting that the importance of the accessory genome in driving bacterial evolution may be highly variable across genera. These results demonstrates that ggMOB can be used to further probe potential genus-genus mobilization dynamics, and thus, provide insight into conjugative mobilization between unculturable bacteria and complex interactions involving multiple genera.

## 2 Results

### 2.1 Overview of ggMOB

The analyses conducted in this paper were derived from an existing resource (Seabolt et al., 2020), which was constructed by curating and annotating 186,887 genomes from NCBI (see MATERIALS AND METHODS). Using the sequence data and annotated features obtained from over 166,000 curated and high-quality WGS datasets, we identified genomes that contained conjugative features (Table 1), which we term conjugative genomes (see MATERIALS AND METHODS). By recording counts of shared features across these conjugative genomes, we constructed ggMOB (the “genus-genus mobilization” network), which contains information about features that are shared between conjugative genus-genus pairs.

**TABLE 1:** Conjugative features included in this study. Color label indicates the color used to represent the conjugative feature in Figure 2. Yellow indicates a conjugative feature defined by a single IPR code and red indicates a family of codes. Genome count indicates the number of genomes that contain the conjugative feature.

Label	Conjugative feature	Genome count	Description
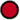	ICE6013	93,267	Includes IS30-like DDE transposase. More closely related to ICEBs1 than Tn5801 Smyth and Robinson. (2009)
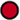	Tn916	59,718	TetM and other resistance genes Clewell et al. (1995)
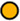	IPR025955	49,841	Type-IV secretion system protein TraC Finn et al. (2016)
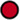	ICEEc2	49,543	set of three genes encoding DNA mobility enzymes and type IV pilus Roche et al. (2010)
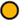	IPR005094	42,760	Endonuclease relaxase, MobA, VirD2 Finn et al. (2016)
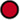	ICEhin1056	42,252	Antibiotic resistance island Mohd-Zain et al. (2004)
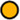	IPR011119	28,752	Unchar. domain, putative helicase, relaxase Finn et al. (2016)
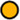	IPR014862	26,295	TrwC relaxase Finn et al. (2016)
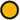	IPR014059	23,368	Conjugative relaxase, N-terminal Finn et al. (2016)
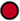	PAPI-1	22,855	Pathogenicity island PAPI-1 of strain PA14.115 gene cluster includes virulence phenotypes Qiu et al. (2006)
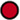	pKLC102	22,460	Hybrid of plasmid and phage origin includes replication, partitioning, conjugation, pili, & integrase genes Klockgether et al. (2004)
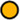	IPR021300	22,284	Integrating conjugative element protein Finn et al. (2016)
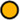	IPR022391	21,465	Integrating conjugative element relaxase, PFGI-1 class Finn et al. (2016)
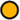	IPR022303	19,664	Conjugative transfer ATPase Finn et al. (2016)
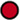	ICEPdaSpa1	19,424	An SXT-related ICE derived; causative agent of fish pasteurellosis Osorio et al. (2008)
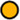	IPR014129	18,029	Conjugative transfer relaxase protein TraI Finn et al. (2016)
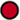	SXT	17,525	Family of conjugative-transposon-like mobile elements encoding multiple AR genes Beaber et al. (2002); Burrus et al. (2006)
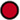	ICEEc1	10,170	High-pathogenicity island (HPI); evidence for Combinatorial Transfers Paauw et al. (2010)
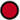	R391	9,916	Archetype of IncJ; carries AR, DNA repair, & mercury resistance genes Böltner et al. (2002)
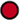	ICEKp1	9,117	Resembles functional ICEEc1 Paauw et al. (2010)
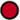	ICESde3396	9,088	Carries genes predicted to be involved in virulence and resistance to various metals Smyth et al. (2014)
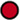	ICEBs1	8,504	Plasmid mobilization and putative coupling protein Lee et al. (2012)
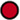	RD2	8,370	Encodes seven putative secreted extracellular proteins Sitkiewicz et al. (2011)
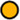	IPR011952	2,640	Conserved hypothetical protein CHP02256 Finn et al. (2016)
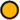	IPR014136	2,050	Ti-type conjugative transfer relaxase TraA Finn et al. (2016)
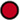	TnGBS2	1,630	See ICE6013 Everitt et al. (2014)
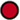	CTnBST	1,520	Tyrosine recombinase family Song et al. (2007)
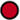	ICEclc	1,465	Cargo for ortho-cleavage of chlorocatechols and aminophenol metabolism (amr genes) Obi et al. (2018)
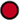	GI3	1,340	Degradation of aromatic compounds and detoxification of heavy metals Lechner et al. (2009)
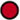	Tn1549	648	VanB-type resistance to glycopeptides with regions Garnier et al. (2000)
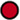	CTn341	389	Encodes tetracycline resistance and its transfer is induced by tetracycline Peed et al. (2010)
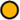	IPR020369	119	Mobilisation protein B Finn et al. (2016)
			(i) excision-integration process Garnier et al. (2000)
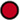	Tn4555	79	Includes cfxA gene encoding
			broad-spectrum beta-lactamase Smith and Parker (1993)
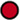	ICESt1	26	Integrative and putative transfer functions Burrus et al. (2002); Bellanger et al. (2009)
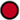	ICEMISymR7A	16	Rhizobial symbiosis genes Ramsay and Ronson (2015)
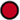	ICESt3	14	Integrative and putative transfer functions Bellanger et al. (2009)
			(ii) vanB2 operon replaces tet(M) Garnier et al. (2000)
			(iii) Conjugative transfer Garnier et al. (2000)
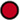	Tn4371	0	Biphenyl and 4-chlorobiphenyl degradation Springael et al. (1993)

### 2.2 Inter- and intra-genus conjugation profiles

Of the 106,443 genomes that contained at least one conjugative feature, 95,781 shared at least one conjugative feature with at least one other genome in the set, indicating a common evolutionary history. These 95,781 conjugative genomes represented close to 47% (631 of the 1,345) of the genera contained in the relational database (Seabolt et al., 2020). The lack of conjugative machinery in the other 714 genera may be a false negative finding (i.e., incomplete list of conjugative features, lack of representation in the utilized NCBI databases, or lack of inclusion in genome assemblies), or could indicate inherent differences in the conjugative ability of genera across the taxonomic tree. Similarly, one might reasonably expect the number of observed conjugative genomes to scale with the number of genomes available for each genus. However, genus representation in NCBI is not uniform across genera, leading to bias in available genomes per genus. To correct for this imbalance, we computed the conjugative genome proportion by normalizing the number of observed conjugative genomes to the total number of genomes per genus (Table 2). This analysis demonstrated that the genera with the largest fraction of conjugative genomes were *not* the genera with the most genomes in NCBI. For example, although *Salmonella* had by far the greatest number of high quality genomes (N = 39,574), it ranked fifth in terms of the proportion of genomes that contained a conjugative feature. The top 30 genera listed in Table 2 all had a conjugative genome frequency greater than 20%, with genomes from the genus *Legionella* containing conjugative proteins over 99% of the time. This high percentage may have been driven by sampling bias in the available NCBI WGS datasets (for example, the *Legionella pneumophila* WGS accessions appear to have been collected from a single site), or it may represent the propensity for conjugation-mediated processes to occur within individual genera.

**TABLE 2:** Proportion of genomes that contained conjugative feature(s), by genus. All genera with over 100 representative genomes are listed, in descending order by the proportion of conjugative genomes in each genus.

Genus	Number of conjugative genomes	Number of total genomes	Proportion conjugative genomes
*Legionella*	1,672	1,686	0.99
*Shigella*	5,423	5,541	0.98
*Klebsiella*	4,682	5,304	0.88
*Elizabethkingia*	102	119	0.86
*Escherichia*	8,140	9,957	0.82
*Stenotrophomonas*	441	563	0.78
*Enterobacter*	894	1,210	0.74
*Vibrio*	2,902	4,017	0.72
*Acinetobacter*	2,621	3,770	0.70
*Pseudomonas*	3,222	4,750	0.68
*Enterococcus*	1,003	1,516	0.66
*Citrobacter*	131	203	0.65
*Salmonella*	24,123	38,808	0.62
*Clostridioides*	1,329	2,183	0.61
*Streptococcus*	8,244	13,766	0.60
*Xanthomonas*	201	357	0.56
*Staphylococcus*	18,034	32,661	0.55
*Rhizobium*	110	202	0.54
*Yersinia*	215	437	0.49
*Lactococcus*	57	117	0.49
*Serratia*	230	619	0.37
*Sinorhizobium*	44	121	0.36
*Bifidobacterium*	137	403	0.34
*Moraxella*	65	192	0.34
*Bacillus*	471	1,471	0.32
*Campylobacter*	5,340	19,501	0.27
*Aeromonas*	80	312	0.26
*Brucella*	230	970	0.24
*Mesorhizobium*	89	385	0.23
*Helicobacter*	118	529	0.22
*Streptomyces*	74	333	0.22
*Corynebacterium*	133	639	0.21
*Neisseria*	153	781	0.20
*Burkholderia*	341	2,053	0.17
*Haemophilus*	65	403	0.16
*Lactobacillus*	144	962	0.15
*Listeria*	848	7,716	0.11
*Clostridium*	40	454	0.09
*Mycobacterium*	1,120	13,129	0.09
*Cutibacterium*	10	118	0.08
*Bordetella*	60	733	0.08
*Chlamydia*	33	496	0.07
Bartonella	3	124	0.02
*Mycoplasma*	2	251	0.01
*Francisella*	0	120	0.00

Next, we identified a total of 5,956 conjugation proteins across genus pairs, i.e., triples of the form (genus1, genus2, protein-name), associated with a total of 23,353,196,048 cargo protein sequences. These results are visualizable as connected nodes in the ggMOB network. This count is non-distinct by protein name as, for instance, Tyrosine recombinase XerD was found both between Salmonella-Salmonella genomes, as well as between Oligella-Proteus genomes and other genera pairs. Of these 5,956 triples, 1,680 contained genome pairs belonging to the same genus (i.e., intra-genus). Some genera were much more likely to contain genomes with conjugative features that matched to genomes from other genera, i.e., inter-genus. For example, we observed over 30 million instances of matching conjugation proteins in genomes from the *Acinetobacter* genus. Of these instances, 84% were to genomes from other genera (i.e., inter-genus), *versus* 16% from genomes within *Acinetobacter* (i.e., intra-genus). Members of the *Acinetobacter* genus are well-known for genome plasticity (Chan et al., 2015), which contributes to important phenotypes such as AMR and biofilm formation.

Genera that shared the highest number of conjugation proteins with *Acinetobacter* genomes included *Escherichia*, *Shigella*, *Vibrio*, *Salmonella*, *Pseudomonas*, *Klebsiella*, *Enterobacter* and *Citrobacter*. While this result was not unexpected given the list of known pathogens contained within these genera, our database of intra- and inter-genus exact-match conjugative features also revealed many unreported and unexpected associations. For example, genomes within the genus Nitrosomonas contained 38,034 instances of conjugative proteins, nearly 100% of which were shared with genomes from the *Shigella* genus. The specific conjugative feature involved in the vast majority of these exact matches was ICEEc2, a relatively recently discovered ICE MGE that was previously shown to transfer competently between *Salmonella enterica* serovar Typhimurium strain and into a *Yersinia pseudotuberculosis* strain. Our results suggest that ICEEc2 has a very broad host-donor range, including many genera that may not yet be described in the literature in reference to this particular ICE.

We found that the likelihood of identifying conjugative features in pairs of genomes within *versus* across genera was highly variable. Of the 36 conjugative features analyzed, 13 were only identified in pairs of genomes belonging to the same genus, i.e., intra-genus (Table 3). However, six conjugative features were more likely to be observed across genera than within genera, i.e., >50% of the observations were inter-genus (Table 3). For example, the exact same CTn341 sequence was observed in pairs of genomes a total of 10,796 times; in 72% of these instances, the pairs of genomes belonged to different genera, indicating a history of inter-genus transfer of CTn341. This conjugative feature belongs to the ICE family of MGEs and plays an important role in tetracycline resistance, and is typically associated with the genus *Bacteroides*, including in most reports related to its functionality (Bacic et al., 2005). However, we observed that 17% of the genome pairs containing exact-match CTn341 sequences belonged to the *Bacteroides* and Alistipes genera, suggesting historical transfer of this important ICE between these genera. Alistipes is an emerging genus with potential health implications (Parker et al., 2020), and the epidemiology and ecology of CTn341 within this genus warrants further investigation. Furthermore, this finding provides additional insight into the potential importance of CTn341 in spreading tetracycline resistance genes across microbial taxa.

**TABLE 3:** Count and proportion of inter- and intra-genus detection of conjugative features.

Conjugative	No. Inter-genus	Prop. Inter-genus	No. of intra-genus	Prop. Intra-genus
Feature	Matches	Matches	Matches	Matches
IPR014059	122,066	0.02	6,098,286	0.98
IPR014862	123,175	0.02	6,246,342	0.98
IPR005094	415,744	0.11	3,224,556	0.89
IPR014136	43	0.00	83,448	1.00
Tn916	110,710,102	0.61	71,585,305	0.39
GI3	74,775	0.20	304,550	0.80
ICEPdaSpa1	8,356,908	0.37	14,101,581	0.63
ICEclc	75,322	0.20	304,722	0.80
ICEhin1056	128,834,838	0.69	58,590,390	0.31
IPR011119	454,906	0.03	16,981,338	0.97
IPR021300	69,832	0.00	20,685,606	1.00
IPR022303	44,341	0.01	7,859,462	0.99
IPR022391	52,638	0.00	14,265,248	1.00
IPR025955	55,3687	0.02	32,073,159	0.98
SXT	17,427,985	0.58	12,525,984	0.42
CTn341	7,760	0.72	3,036	0.28
ICEEc1	1,107,736	0.18	5,010,865	0.82
ICEEc2	28,902,519	0.33	58,830,977	0.67
ICEKp1	1,629,343	0.20	6,550,799	0.80
IPR011952	50	0.00	3,154,129	1.00
IPR014129	75,786	0.02	4,557,985	0.98
R391	796,084	0.10	7,476,937	0.90
Tn4555	972	0.72	382	0.28
pKLC102	10,135	0.00	5,277,724	1.00
IPR020369	577	0.12	4,406	0.88
Tn1549	866	0.04	23,336	0.96
ICESde3396	7,659	0.00	1,676,016	1.00
CTnBST	337,318	0.63	196,675	0.37
ICEBs1	0	0.00	1,220,869	1.00
ICE6013	46,112	0.00	406,783,868	1.00
ICESt1	31	0.12	237	0.88
ICEMISymR7A	0	0.00	37	1.00
PAPI-1	0	0.00	6,101,539	1.00
ICESt3	0	0.00	97	1.00
RD2	0	0.00	1,547,322	1.00
TnGBS2	0	0.00	56,243	1.00

### 2.3 Cargo gene profiles

To characterize the set of genes that are most likely to have been associated with conjugative HGT events, we identified all proteins that were contained in at least two conjugative genomes with 100% sequence identity. Out of 51,362,178 total unique protein sequences in the source database, 28,042 were identified as conjugation-associated proteins (i.e., conjugation machinery), and 11,276,651 were identified as candidate cargo proteins. The full set of cargo proteins mapped to 20,550 distinct names (excluding “putative protein(s)” or “hypothetical protein(s)”), with a wide range of frequencies within and between genera. Annotation of the conjugative genomes demonstrated that the vast majority of conjugative and cargo proteins were adjacent within the genome (Figure 2). Moreover, within each genome, the conjugative features were more likely to be proximate to putative cargo protein *versus* non-cargo proteins, suggesting again a common evolutionary history.

### 2.4 Genotypic AMR and association with conjugation features

A subset of the observed cargo protein names were associated with a set of confirmed AMR protein names. We identified this subset by selecting only those names that Prokka assigned to sequences mapping to a name defined in MEGARes v1.0 (Lakin et al., 2016), a comprehensive AMR database. The entity relations in our database ensured a 1:1 mapping between gene and protein names and their respective sequences. Of the 3,824 distinct sequences contained in MEGARes, Prokka identified 3,674 as valid sequences coding for protein. These 3,674 distinct proteins were assigned 286 distinct names, excluding “putative protein” and “hypothetical protein”. These unnamed proteins comprised just 1% of the MEGARes protein set. While this highly curated set is certainly not a comprehensive list of all proteins contributing to AMR, it is an initial set to estimate the fraction of AMR proteins within the larger set of conjugation and cargo proteins.

To further investigate the microbial genomics of AMR in relation to HGT events, we used plasmid sequences from NCBI to identify the set of AMR-specific cargo proteins found in a plasmid sequence. We then compared the distribution of AMR proteins in plasmids *versus* conjugative genomes (Figure 3). The distribution of AMR genes differed between plasmids and conjugative genomes, with a high probability that AMR genes were identified in conjugative (*versus* non-conjugative) genomes, and a much lower probability of being identified in plasmids (Figure 3). While plasmids are often critical to the microbial ecology of AMR, this analysis suggests that other conjugative processes may drive the vast majority of AMR gene exchanges between bacteria. This dynamic has been reported for specific AMR gene groups, including carbapenem AMR genes (Botelho et al., 2020), and also supports previous analyses of conjugative machinery across bacterial genera (Guglielmini et al., 2011).

To analyze the distribution of AMR proteins across genomes, we calculated the probability that each AMR gene was identified in conjugative *versus* non-conjugative genomes (Table 4). From the complete list of 286 AMR protein names, 220 were found more often in conjugative genomes; three were found with equal probability in conjugative and non-conjugative genomes; and 63 were found more often in genomes that did not contain any conjugation proteins. Given that the proportion of conjugative genomes in our database was 51%, these results suggest that AMR genes are disproportionately represented within conjugative genomes.

**TABLE 4:** Probability of observing AMR proteins in genomes that also contained conjugative features.

AMR protein name	Number of Genomes	Number of Conjugative Genomes	Proportion Conjugative Genomes
Rob DNA-binding transcriptional activator	4,559	4,559	1.00
Transposon Tn10 TetD protein	4,559	4,559	1.00
Tetracycline resistance gene Tet(M)	441	441	1.00
Outer membrane protein YedS	288	288	1.00
Regulator of RpoS	288	288	1.00
Beta-lactamase Toho-1	319	318	1.00
AcrAD-TolC permease subunit	1,584	1,576	0.99
Multidrug efflux pump subunit AcrB	1,584	1,576	0.99
DNA topoisomerase subunit A	1,091	1,085	0.99
MATE family multidrug efflux pump protein	1,607	1,596	0.99
Carbapenem-hydrolyzing beta-lactamase KPC	1,914	1,899	0.99
Beta-lactamase OXA-1	1,188	1,175	0.99
Inner membrane protein HsrA	350	346	0.99
Putative transport protein YdhC	342	338	0.99
Aclacinomycin methylesterase RdmC	419	414	0.99
Beta-lactamase OXA-2	199	196	0.98
Chloramphenicol efflux MFS transporter CmlA1	795	783	0.98
Beta-lactamase OXA-10	521	513	0.98
armA*	994	978	0.98
rRNA large subunit methyltransferase H	4,087	3,989	0.98
Multidrug resistance operon repressor	486	67	0.14
Outer membrane protein OprM	478	65	0.14
Methicillin-resistance regulatory protein MecR1	8,344	1,007	0.12
Phosphoethanolamine-lipid A transferase	8,344	1,007	0.12
HTH-type transcriptional repressor BepR	467	55	0.12
Bifunctional polymyxin resistance protein ArnA	507	53	0.10
Methicillin resistance regulatory protein MecI	6,583	395	0.06
Metallothiol transferase FosB	6,584	395	0.06
Multidrug efflux transporter MdtL	419	9	0.02
Multidrug efflux pump subunit AcrA	420	7	0.02
HTH-type transcriptional regulator SyrM 1	414	3	0.01
RND transporter permease subunit OqxB3	358	2	0.01
Aminoglycoside 2′-N-acetyltransferase	9,723	5	0.00
DNA-binding response regulator MtrA	9,854	2	0.00
Putative acetyltransferase	5,232	1	0.00
Quinolone resistance protein NorB	213	0	0.00

Only AMR proteins that appeared in more than 100 genomes were considered; and only AMR proteins that occurred in conjugative genomes with a probability ≥0.98 or ≤0.15 are listed in this table. Full data available in the Supplementary Table S3. *Full protein name: 16S rRNA (guanine(1405)-N(7))-methyltransferase.

The above analysis does not distinguish between different types of conjugation features, and also treats AMR proteins as independent features. However, the data in Figure 2 demonstrate that many of the conjugation features defined in Table 1 often co-occur in the same genome, as do some of the AMR proteins. To gain insight into these correlations, and to identify groups of AMR proteins associated with different conjugation families, we performed a genomic co-occurrence analysis across all conjugation features for the 138 AMR proteins found in conjugative genomes with a frequency of at least 5 times compared to identification in non-conjugative genomes (i.e., these AMR proteins were highly represented in conjugative genomes, Figure 4). The results of this analysis demonstrated that some conjugation features frequently co-occurred within genomes with both other conjugation features as well as multiple AMR proteins. For example, IPR005094 co-occurred with the highest diversity of AMR protein names (N = 139, see Supplementary Table S2). Many co-occurrence patterns reflected known biological associations. For example, the Tn916 conjugation feature co-occurred most frequently with tetracycline ribosomal protection protein TetM, a genomic association discovered over 3 decades ago (Su et al., 1992). While TetM seemed to co-occur with a few select conjugation features (such as Tn916), other AMR protein names co-occurred with many conjugation features. For example, many of the AMR names associated with extended-spectrum beta-lactam and carbapenem resistance (e.g., beta-lactamases Toho-1, OXA-1, OXa-2, OXA-10, SHV-2, and KPC) co-occured with the majority of evaluated conjugation features, which may provide partial explanation for the observed rapid expansion of these important AMR genes within Enterobacteriaceae (Logan and Weinstein, 2017). Similarly, the recently widely-publicized mcr-1 protein co-occurred with multiple conjugation features, which both strengthens and expands upon recent findings that this AMR gene has been mobilized on numerous plasmid types (Wang et al., 2018). Co-occurrence data such as those provided in Figure 4 may represent a new and sustainable (i.e., easily updated) source of information regarding the potential for new and emerging AMR genes to expand within and across bacterial populations *via* HGT. This information, in turn, could help to prioritize and focus public health and human clinical decision-making regarding AMR.

Conversely, 29 AMR proteins occurred at least 5x more frequently in non-conjugative genomes compared to conjugative genomes (Supplementary Figure S1). Of note is the observation that no beta-lactam AMR protein names occur in this list of 29 AMR names, which contrasts starkly to the preponderance of beta-lactam-associated AMR names in Figure 4, again suggesting that beta-lactam resistance is tightly coupled with conjugative machinery, and that conjugation-mediated exchange is the primary evolutionary driver of beta-lactam resistance. By comparison, several mechanisms of multi-drug resistance (MDR) are contained within the list of 29 AMR proteins observed more frequently in non-conjugative *versus* conjugative genomes, i.e., AcrB, AcrE, OqxB7, mdtA, mdtE, mdtH, and MexB. These mechanisms of MDR tend to be multi-function, i.e., the proteins confer multiple functional benefits to bacteria, in addition to AMR. Together, the results of Figure 4 and Supplementary Figure S1 suggest that proteins with more specific AMR functions tend to be disproportionately represented amongst conjugative genomes, while more generalist proteins tend to be disproportionately represented within non-conjugative genomes. One hypothesis for this observation is that the fitness cost-benefit dynamics differ for generalist *versus* specialist genes, such that specialized genes are more likely to transiently yet rapidly spread within bacterial populations *via* the so-called ‘accessory genome’ (which includes conjugation-mediated exchange), whereas generalist genes are more likely to be maintained permanently within bacterial genomes, and thus are less likely to be identified as conjugation-associated cargo.

### 2.5 Phenotypic AMR and association with conjugative genomes

Given our hypothesis that conjugation-mediated spread of specialized AMR genes may be promoted by more specific evolutionary pressures such as antimicrobial drug exposures, we hypothesized that this signature of selective pressure may also manifest in the phenotypic properties of conjugative *versus* non-conjugative genomes. To evaluate this, we queried the NCBI BioSample assay metadata in our relational database, to identify isolates that had been subjected to phenotypic antibiotic susceptibility testing (AST) to known antibiotic compounds. For the 186,887 highest quality genomes, the NCBI assay metadata contained 15,286 phenotypically-confirmed resistant genome-compound AST results, representing 13,076 tests for conjugative genomes and 2,210 tests for non-conjugative genomes. Altogether, 1,242 genomes were used in these tests, of which 1,023 were conjugative genomes and 219 were non-conjugative genomes. For each antibiotic compound listed in the AST results, we computed the number of phenotypically resistant isolates with conjugative-vs. non-conjugative genomes.

The results of this analysis revealed that phenotypic resistance occurred in conjugative genomes with probability >80%, regardless of compound being tested (Table 5 and Supplementary Table S3). As with the disproportionate representation of AMR proteins within conjugative genomes, the phenotypic AMR data suggest that microbial AMR dynamics are driven largely by conjugation-mediated processes. However, it is also important to note that NCBI phenotypic assay data is likely biased due to the motivations for clinicians and researchers to submit isolates for phenotypic testing. Therefore, to test for SRA sampling bias with respect to these compounds, we also measured the phenotypically-resistant fraction expected for *randomly selected* genomes, based on the number of genomes tested per compound in Table 5 and the number of conjugative and non-conjugative genomes across the entire database. This null hypothesis was tested by running 100 bootstrapped trials for each compound (Table 3). The observed average probability that phenotypic AMR was expressed by an isolate with a conjugative genome was 0.85 ± 0.05 *independent of antibiotic compound*, i.e., weighted by total genomes tested per compound (Table 5). In a random process, the probability would be expected to be near 51% given the fraction of all genomes with conjugation features. These results further support the importance of conjugation in the microbial ecology and epidemiology of both genotypic and phenotypic AMR.

**TABLE 5:** Associations between phenotypic AMR and representation of conjugative *versus* non-conjugative genomes.

Antibiotic	Number of Resistant Genomes	Number of Resistant Conjugative Genomes	Expected Number of Resistant Conjugative Genomes^*^	Observed Proportion Conjugative Genomes
doripenem	215	207	86 ± 7	0.96
cefepime	272	259	108 ± 8	0.95
ampicillin-sulbactam	433	402	173 ± 10	0.93
imipenem	429	396	171 ± 11	0.92
piperacillin-tazobactam	280	258	112 ± 9	0.92
meropenem	402	367	161 ± 11	0.91
trimethoprim-sulfamethoxazole	868	775	348 ± 14	0.89
ertapenem	222	197	89 ± 8	0.89
levofloxacin	741	644	297 ± 14	0.87
gentamicin	813	706	325 ± 13	0.87
ciprofloxacin	915	794	367 ± 14	0.87
amoxicillin-clavulanic acid	277	240	111 ± 8	0.87
ceftriaxone	984	849	394 ± 16	0.86
tetracycline	700	603	281 ± 12	0.86
ceftazidime	852	731	342 ± 14	0.86
cefotaxime	889	758	355 ± 15	0.85
tobramycin	674	574	270 ± 12	0.85
ampicillin	1042	852	418 ± 16	0.82
amikacin	383	313	154 ± 10	0.82
aztreonam	989	805	397 ± 15	0.81
cefazolin	1002	813	402 ± 16	0.81
cefoxitin	757	608	304 ± 15	0.8
nitrofurantoin	713	559	287 ± 12	0.78
cefotetan	124	95	49 ± 6	0.77

^*^Only drug compounds with 100 or more non-redundant resistant genome measurements and >76% representation in conjugative genomes are listed. The expected number of conjugative genomes was estimated based on a bootstrapped random selection process with 100 trials (null hypothesis), using the number of assays and the actual fraction of genomes with conjugative features (˜51%).

### 2.6 Genome clustering by conjugative feature profile

The specific proteins transferred between bacteria are known to vary by conjugation feature, for example as demonstrated by the co-occurrence patterns of conjugation features and AMR proteins within genomes (Figure 4 and Supplementary Figure S1). To demonstrate the structuring of bacterial populations by conjugation-cargo co-occurrence patterns, we generated the same co-occurrence matrix for all cargo proteins and all conjugative features within genomes that contained a high frequency of cargo proteins (Figure 5). We then calculated the genome-genome Euclidean distance for all genomes in the co-occurrence matrix, using a vector of normalized conjugation features (see *Methods*, Supplementary Figure S2). The results demonstrate clusters of genomes with similar conjugative features. While these clusters often reflect classical bacterial taxonomic structure, there are also instances of discordance between the clustering based on conjugation profile and traditional grouping based on taxonomy. These results reflect the evolutionary dynamics of bacterial populations, and suggest that the relative importance of vertical *versus* horizontal gene transfer events is highly variable. Such variability in the importance of HGT events has been previously reported, including differences in plasmid *versus* ICE-mediated exchange and interactions with bacterial host range (Cury et al., 2018). This finding has far-reaching and complex implications for applications that rely on a measure of phylogenetic relatedness, i.e., outbreak detection and source attribution. In some cases the use of the core genome may be sufficient to accurately reflect bacterial phylogenetic relatedness; while in others, the information in the core genome may obfuscate the true relationships between bacterial isolates. As WGS data become more widely used, these complexities must be considered, and in some cases, incorporated into phylogenetic analysis workflows.

The relational database underlying this work and the ggMOB tool is a necessary (yet not sufficient) component of improved interpretation and use of WGS data. We note that, in principle, one can use the co-occurrence matrix and vectorization procedure on genomic properties other than conjugative features. This provides a flexible and customizable approach for defining a different set of genomes of interest within the *mobilome*, which can then be used to (re)classify organisms not by name, but by genome-genome distance in a space of mobilization features. This capability could represent a powerful tool for improving our understanding of bacterial evolution while also informing the next generation of applied WGS computational and statistical pipelines.

## 3 Discussion

The public availability of large scale genomic data makes it possible to apply cloud computing technology and big data techniques to study important phenomena in molecular and microbiology. Curating these data in a relational database with biologically structured entity relations (i.e., linking genomes, genes, proteins, domains, and metadata) provides a powerful method with which to ask biological questions about the data. We leveraged this approach in the current study of cargo and conjugation, which is an essential mechanism by which bacteria acquire new phenotypes, transmit molecular functions, and adapt to stress. Furthermore, these events are critical for understanding bacterial evolution and phylogeny (Guglielmini et al., 2011; Abby et al., 2012; Bellanger et al., 2014). Our work not only sheds light on conjugation-mediated cargo transfers between and within genera, but also demonstrates the ability of mining and analyzing large datasets in improving our understanding of bacterial evolutionary dynamics. The network of putative genus-genus conjugation features and candidate cargo genes can be dynamically visualized using the ggMOB tool, which supports hypothesis generation and testing related to intra- and inter-genus conjugation dynamics.

In our analysis we identified sets of proteins with the strongest evidence as conjugation and cargo proteins. This was accomplished by selecting only those proteins that exhibited both 100% sequence identity and co-occurrence in pairs of genomes containing identical conjugation-associated sequences. With this strict selection process, the putative cargo proteins exhibited a high degree of spatial correlation within assembled contigs (i.e., they were highly adjacent to each other, as well as to the conjugation protein itself). Other proteins in these genomes may also have been transferred (or are transferable) by bacterial conjugation, but they did not meet our strict selection criteria. Considering only strictly-selected candidate cargo proteins, we were able to profile the frequency of conjugation-mediated protein exchange within and between genera.

Our results suggest that conjugation-mediated exchange is not uncommon, affirming prior studies (Guglielmini et al., 2011; Bellanger et al., 2014). Conjugation-related proteins were observable in 51% of bacterial genomes and in 631 of 1,345 genera (approximately 47%). Frequency of intra- and inter-genus conjugation-mediated exchange varied significantly depending on the taxa involved, suggesting that taxonomy greatly influences genetic exchange of, e.g., AMR or pathogenicity proteins (Delavat et al., 2017). By quantifying this across a large database of high-quality WGS data, we measured the “exchange likelihood” between different genera. These likelihoods can be visualized dynamically in the ggMOB tool, which reveals distinct clusters of genera that share conjugative features with exact sequence match. This suggests that the likelihood of protein transfer varies substantially by genus pair, and that the bacterial composition within a given environment is an important consideration when attempting to evaluate mobilization potential within a microbial community (Lopatkin and Collin, 2020; Neil and Allard, 2021).

While we have conducted this analysis for a specific set of conjugation features (Table 1), the analytic approach can be applied to any MGE(s) and cargo protein(s) of interest. As such, our overall approach represents a method for obtaining a long-range evolutionary view of transfer likelihood between diverse bacterial taxa, including pathogens and commensal bacteria (Guglielmini et al., 2011). These baseline exchange likelihoods are critical parameters for risk analysis at the microbial community level, including for applications such as personalized microbiome medicine, and microbiome-centric surveillance.

Bacterial taxon is not the only significant driver of exchange likelihood; we also observed that putative, successful transfer events were more likely to involve cargo proteins that infer fitness advantage to the involved bacterial populations, such as AMR. While any gene can, in principle, be transmitted as a cargo gene in conjugative exchange, only a subset of transferred proteins will increase the fitness of the receiving organism. The likelihood of observing successful transfer depends on a large number of factors including the environment, the existing proteins in the recipient chromosome, the cargo proteins themselves, and the survival probability of the organism (Cohen et al., 2010).

Conjugation-mediated protein transfer that improves fitness may increase survival probability. Therefore, chromosomal arrangements that group *fitness-conferring* cargo proteins near the conjugation machinery will be observed more frequently than those arrangements that involve neutral or disadvantageous proteins. Conversely, very common proteins that aid in stress response may be less likely to be transferred as cargo, since the relative fitness advantage is diminished for proteins that are already likely to be present within a bacterium (i.e., proteins that confer redundant function). The particular stressor—as well as the specific advantageous proteins of interest—depend on phenotype of interest. This view is exemplified by the data in Table 4 which shows that rare AMR proteins are more likely to be found as cargo in genomes that also contain conjugative proteins, as compared to genomes that do not. Conversely, common AMR proteins are less likely to be found in genomes that contain conjugative machinery. One might hypothesize that, with chromosomal rearrangement, nature effects a real world experiment to dynamically optimize cargo protein collections—thereby spreading rare (but useful) proteins and gene combinations over time.

**FIGURE 1:**
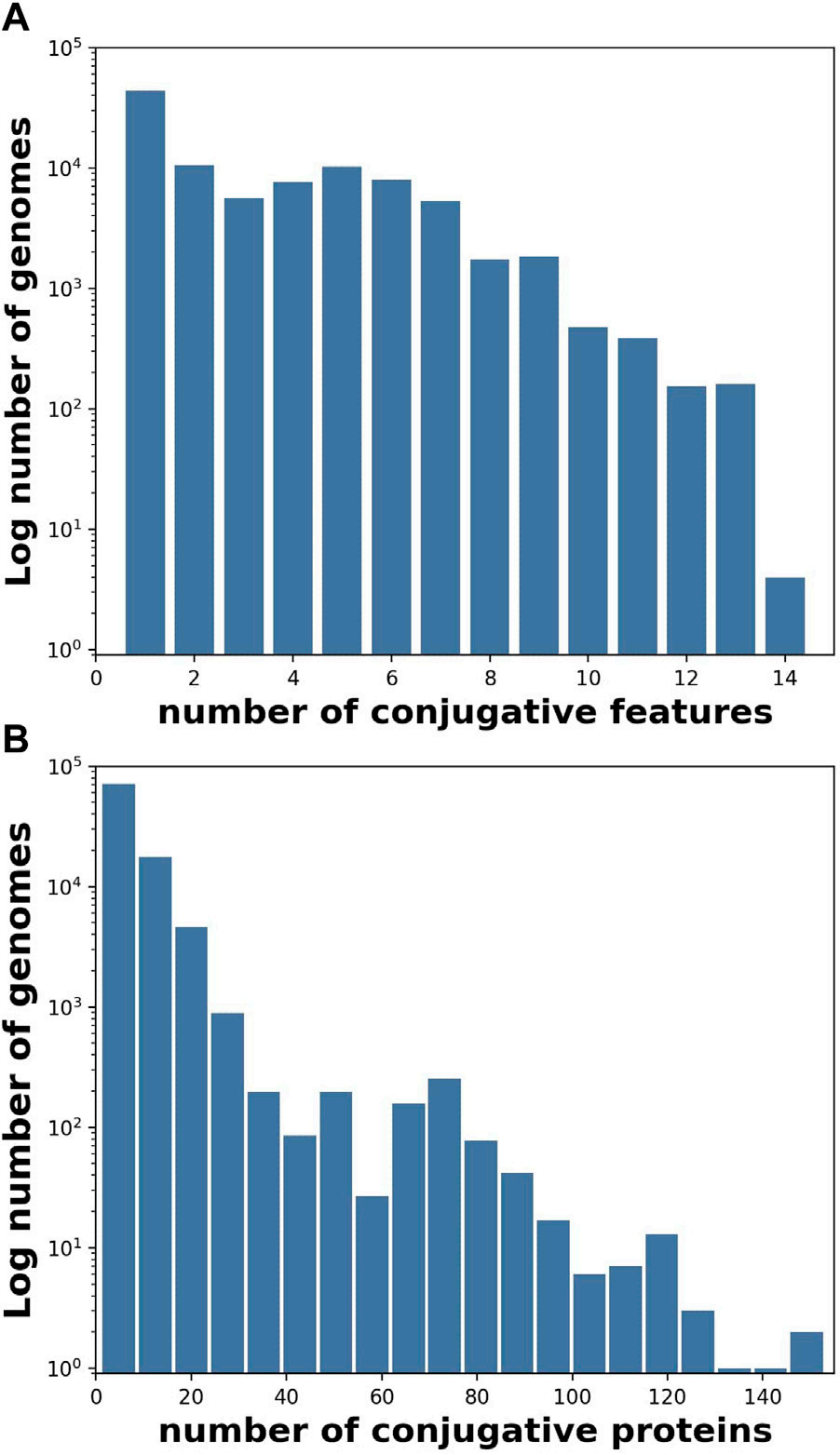
Individual genomes typically contained more than one conjugative feature (Table 1), and often contained more than one protein per feature. **(A)** Histogram (log frequency) of the number of conjugative features per genome, and **(B)** Histogram (log frequency) of the number of conjugative proteins per genome for all 106,433 genomes containing at least one conjugative feature.

**FIGURE 2:**
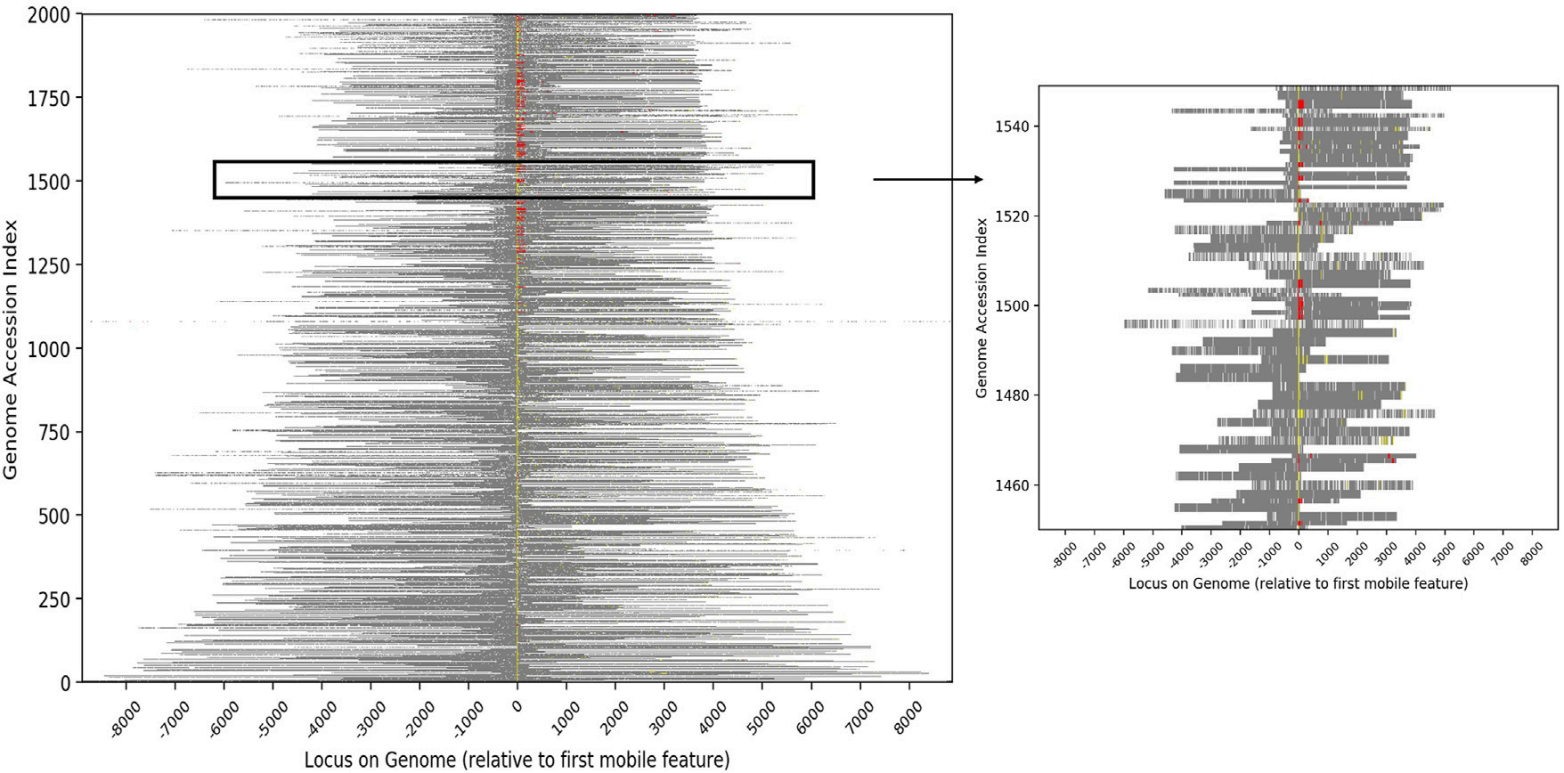
Heatmap showing the relative genomic positions of conjugative features and putative cargo proteins for the 2,000 genomes with the greatest number of cargo proteins. Conjugative features are represented as color pixels based on the colors shown in Table 1, with yellow representing proteins assigned specific IPR codes and red representing protein families from the literature. Cargo proteins are shown in grey and other chromosomal DNA in white. Each genome is bit shifted to the left until the first conjugative feature is centered in the figure. Most genomes contained more than one conjugative protein (all contain at least one). The inset highlights the genomes at indices 1450–1550 in order to expand a subset of the data.

The particular cargo proteins shared between chromosomes varied by conjugation feature, as demonstrated by the AMR proteins analyzed in Figures 1, 4. Considering all conjugation features used in this study, our results suggest that conjugation dynamics are important in structuring genomic content, and thus driving phylogenetic evolution. Based on Figure 5, it seems that sometimes these evolutionary conjugation dynamics can sometimes overpower other taxonomic drivers, such that genus-level genomes do not always cluster together. To demonstrate this conjugation-driven phylogeny, we used the data in Figure 5 to generate Figure 2, which represents the distance between all pairs of genomes based on Euclidean distance between their representations as normalized conjugation feature vectors. The resulting hierarchical clustering shows that the dominant conjugation features are represented in genomes across different genera and, conversely, that individual genera include genomes with differing conjugation profiles. This abrogation of genus-level taxonomy due to conjugation-related genomic content is an inevitable consequence of the inter-genus transfers visualized in ggMOB. Given the reality of conjugative exchange, there is no reason to expect that taxonomic classification by organism name will always predict the composition of conjugation-associated cargo proteins. However, by selecting genomes based on a particular phenotype of interest, it is possible to classify organisms and genome-genome distances based on a feature space defined by conjugation (or other mobilization) proteins, as in Figure 2. Given the ubiquity and diversity of conjugation and other types of HGT (Guglielmini et al., 2011), these types of genome clustering techniques may provide crucial information about bacterial evolution that is not contained within traditional phylogenies. In this regard, the ability to filter the ggMOB data based on conjugation features of interest may particularly useful.

## 4 Materials and methods

### 4.1 Creation of relational database

Here we briefly describe the process used to create the relational database that underlies ggMOB; further details can be found at (Seabolt et al., 2020). First, we downloaded whole genome sequences from NCBI’s Reference Sequence Database (RefSeq), which we then filtered to only obtain genomes that were identified as being of bacterial lineage, and as having an assembly level of “Complete Genome”. We added to this set of genomes non-assembled sequence data from NCBI’s Short Read Archive (SRA) by first downloading all datasets that had the following criteria: (1) the data consisted of WGS data generated from bacteria, as defined according to their taxonomic lineage; (2) the data were Illumina short-read sequence data from DNA; and (3) the sequence data were paired-end. Long-read and transcriptomic data were not considered. We note that we downloadeded all the SRA data in FASTQ format using the SRA toolkit (Sugawara and Shumway, 2010), and assembled them into contigs using SPAdes (Bankevich et al., 2012). We discarded any genome assemblies that contained more than 150 contigs (of size >500 bp) or had an N50 of less than 100 kbp. We note that only 48% of bacterial genomes met the aforementioned curation thresholds from the original corpus of SRA datasets. Next, we eliminated any assembled genomes in which a significant proportion of *k*-mers originated from multiple genomes across different genera. This removed another 13,044 genomes from consideration. This last step ensured all the genomes used for analysis were from a pure single bacterial isolate with valid genus-level classification, hence minimizing the probability of contamination. We obtained a total of 159,628 genome assemblies after filtering for all the above criteria, and a total of 186,887 genomes when including the genome assemblies from RefSeq.

Next, we annotated all the genomes using Prokka 1.12 (Prokka, 2014), resulting in the collation of the genome, gene, and protein data entities into CSV files. After annotation, we determined the protein domains using InterProScan 5.28–67.0 (Quevillon et al., 2005) with all available analyses provided by InterProScan (16). This resulted in 16 JSON files that were then parsed to create a set of CSV files. The annotation process yielded a total of 66,945,714 unique gene sequences; 51,362,178 unique protein sequences; and 138,327,556 unique protein domains along with related functional annotations.

Using the curated data, we created a relational database using IBM’s DB2 system, which contained the following five different entity types: genomes, genes, proteins, protein domains, and functional annotations. These entities were determined by the above data curation, assembly and annotation steps; each entity in the database was stored using the MD5 hash of the sequence itself to create a unique identifier. Thus, we can quickly query for an entity within the database using the unique identifiers as a key. We stored the relations between entities as tables in the relational database, e.g., the genes corresponding to a particular genome in a table. We note this saves storage because unique sequences are stored only once in their respective tables and the tables point to all the parent entities in which they are found. Thus, while database construction required 1468 CPUs, 6TB RAM, and 160 TB of hard drive space, the final relational database scales efficiently with the addition of new sequences (Seabolt et al., 2020).

### 4.2 Creation of ggMOB

After curation and annotation of the data, we identified all candidate conjugative and cargo proteins in order to create ggMOB. In particular, we used both the primary literature and the InterProScan coding system to generate a list of conjugative features for ggMOB. This led us to consider the following 12 InterProScan codes:

1. IPR005094 describes relaxases and mobilisation proteins, as exemplified by MobA/VirD2 (Pansegrau et al., 1993; Byrd and Matson, 1997; Quevillon et al., 2005).
2. IPR011119 represents a domain found in Proteobacteria annotated as helicase, conjugative relaxase or nickase (Street et al., 2003; Quevillon et al., 2005).
3. IPR014059 codes for a domain in the N-terminal region of a relaxase-helicase (TrwC) that acts in plasmid R388 conjugation. It has been associated with both DNA cleavage and strand transfer activities, and members of this family are frequently found in genomic proximity to conjugative proteins thought to indicate the presence of integrated plasmids when identified in bacterial chromosomes (Quevillon et al., 2005; Boer et al., 2006).
4. IPR014129 represents proteins in the relaxosome complex, exemplified by TraI, which mediates the single-strand nicking and ATP-dependent unwinding of the plasmid molecule *via* two separate domains in the protein (Matson and Ragonese, 2005; Quevillon et al., 2005).
5. IPR014862 represents a conserved domain found in the relaxosome complex, as exemplified by TrwC (Quevillon et al., 2005; Boer et al., 2006).
6. IPR021300 represents a conserved domain observed in ICE elements in the protein family PFL_4695, and originally identified in *Pseudomonas fluorescens* Pf-5 (Quevillon et al., 2005; Mavrodi et al., 2009).
7. IPR022303 describes a family of conjugative transfer ATPases representing predicted ATP-binding proteins associated with DNA conjugative transfer. They are found both in plasmids and bacterial chromosomal regions that appear to derive from integrative elements such as conjugative transposons.
8. IPR025955 describes a family of TraC-related proteins observed in Proteobacteria. TraC is a cytoplasmic, membrane protein encoded by the F transfer region of conjugative plasmids, and is required for the assembly of the F pilus structure, which creates and maintains contact between the donor and recipient cells during conjugation. The family includes predicted ATPases associated with DNA conjugative transfer (Schandel et al., 1992; Quevillon et al., 2005).
9. IPR022391 represents the N-terminal domain of proteins associated with conjugative relaxases in the PFGI-1 class, which includes TraI putative relaxases required for ICE function. While these relaxases are similar in function to TraI relaxases of the F plasmid, they have no sequence homology (Quevillon et al., 2005).
10. IPR011952 represents CD-NTase-associated protein 3, a group of proteins that function as part of CBASS (cyclic oligonucleotide-based antiphage signaling system), which provides immunity against bacteriophages (Millman et al., 2020).
11. IPR014136 encompasses TraA, a Ti-type conjugative transfer relaxase that likely nicks the OriT site and unwinds the coiled plasmid prior to conjugative transfer (similar to TraI(F) in this respect) (Harris et al., 1999).
12. IPR020369 represents mobilization protein B (MobB), which is thought to play a role in conjugative exchange by presenting MobA and its covalently-linked plasmid DNA to the conjugative pore for subsequent export (Meyer, 2011).

We supplemented the IPR features with additional conjugative features that provide essential functions in the conjugation process, including conjugative relaxases, nickases, helicases, and other mobilisation proteins (Schandel et al., 1992; Pansegrau et al., 1993; Byrd and Matson, 1997; Boer et al., 2006) (Table 1). These conjugative features contain substantial sequence diversity, (Frost et al., 2005; Wozniak and Waldor, 2010; Perry and Wright, 2013; Johnson and Grossman, 2015; Singer et al., 2016), but also represent conserved domains involved in the machinery required for conjugative transfer. Using standard SQL queries, we obtained a list of the unique identifiers that have one or more of the features described above, resulting in a list of 28,042 candidate conjugation proteins. From this list, we removed those that appeared exactly in only a single genome, which further reduced the list of potential conjugation proteins to 15,398 across 95,781 genomes. We refer to this resulting set of genomes as *conjugative genomes* because they contain putative conjugation features (Supplementary Table S4). We note that these genomes represent 51% of all genomes in the relational database.

Next, we performed SQL queries in order to identify proteins most likely to be cargo based on evidence of conjugative transfer. To minimize misclassification of proteins as cargo, we applied two rules: (1) the proteins had to be present in at least two genomes with the same conjugative protein; and (2) they could not be present in any non-conjugative genome. To accomplish the query, we first queried the database for all proteins in the 95,781 conjugative genomes, which produced a list of 387,682,038 distinct < conjugative genome accession number, protein > tuples. In many cases a unique sequence was observed in more than one genome, and therefore, in total there were 21,207,794 distinct protein sequences in the set of conjugative genomes. Next, we filtered this list in order to identify the set of proteins that appeared in two or more conjugative genomes. To further reduce false positive identification of transfer by conjugation (vs. being vertically transferred), we discarded any protein that appeared in any of the 99,052 non-conjugative genomes. With this strict selection process, we identified 11,276,651 distinct sequences that we refer to as *cargo proteins*, i.e., proteins with the greatest evidence of conjugative transfer. Lastly, we tabulated these results to produce a list of triples of the form < cargo protein, conjugative genome A, conjugative genome B> which describe genome A and genome B contained at least one identical conjugative protein sequence, yielding a total of 4,938,737,476 putative transfers. We used this file as input to a custom python script that identifies all intra- and inter-genera cargo protein transfers for each protein by comparing all pairs of genomes in order to identify the intersection of conjugative proteins of each pair of genomes, and the cargo proteins (if any) in this intersection. The output of this script was used to create the ggMOB network, which contains a node for each genus, and an edge between any pair of nodes in which the value of co-occurrence of <conjugative protein, cargo protein> is non-empty (see https://github.com/Ruiz-HCI-Lab/ggMOB for source files and code).

### 4.3 Additional analyses

#### 4.3.1 Conjugation and cargo gene proximity

To characterize the genomic proximity of conjugative and cargo genes within the conjugative genome pairs, we used our compiled list of genera, genomes, conjugative and cargo proteins, along with Prokka’s accession index, which indicates the position of a gene or protein sequence within the assembled sequences. While this approach was limited by the fact that the order of assembled sequences is unknown, the Prokka index does indicate position of annotated sequences within each assembled sequence; this information was used in a visual display of genomic distance (Figure 2).

#### 4.3.2 Characterizing AMR genes

We identified all AMR proteins by querying our relational database with all sequences in the MEGARes database (Lakin et al., 2016). To obtain consistent annotations, we annotated the sequences in MEGARes in the same format as was used to annotate the set of all proteins in the database. We note that we were able to maintain high confidence that these annotations represent AMR proteins because the annotations were derived by exact sequence matching. Next, we extracted the set of cargo proteins with (self-consistent) names that matched any MEGARes AMR protein name. These data were then used to compute the frequency of observing each AMR gene in genomes that contained and did not contain conjugative features.

#### 4.3.3 NCBI antibiotic susceptibility testing BioSample data

To analyze associations between phenotypic AMR and genomic conjugation features, we retrieved metadata for each NCBI accession that contained antimicrobial susceptibility testing that contained AST data, which include genomic accession number for each isolate, the antibiotic compound(s) against which it was tested, the AST type, and the phenotypic outcome (resistant, susceptible, or intermediate). We only considered those isolates with a resistant phenotypic outcome to be resistant. By linking the BioSample accession with the SRA accessions in our relational database, we were able to identify genomes for which corresponding AST data were available. These genomes were used in our analysis of phenotyic AMR and conjugative features.

#### 4.3.4 Classification of plasmid and conjugation proteins

**FIGURE 3:**
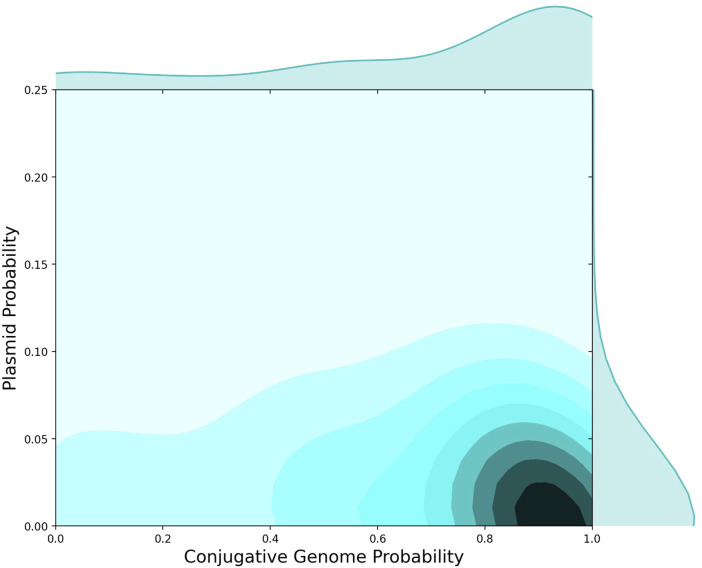
AMR Protein Detection in Plasmids versus Conjugative Genomes. 2D histogram showing the probability that AMR proteins were found in conjugative genomes (*x*-axis) *versus* the fraction of AMR proteins observed on plasmids (*y*-axis). Independent of presence on conjugative genomes, 5%–10% of AMR proteins were observed on plasmids, whereas the majority of AMR proteins were found in conjugative genomes.

We note that many of the InterPro codes listed in Table 1 contain conjugative machinery found in both plasmids and ICEs. Since these two groups of MGEs exhibit unique microbial ecological and epidemiological dynamics regarding AMR, we attempted to further annotate conjugative proteins identified within our set of genomes. To accomplish this, we first queried NCBI for bacterial plasmids. All of these assembled bacterial plasmids were downloaded from NCBI and annotated *via* our Prokka and InterProScan pipelines. The annotated plasmid associated proteins were then placed in a database table. The MD5 hash was used as the primary key for entries in this table, as was true for every sequence entity in the FGP database. Determining whether a protein had been observed on a plasmid in any of the reference genomes was then accomplished by querying for the primary (i.e., FGP) key of each protein within the table of plasmid-associated proteins. If the primary key existed in both tables, we considered that protein to be a plasmid-associated protein. These data were used in the analyses shown in Figure 3.

#### 4.3.5 Tabulation of data

**FIGURE 4:**
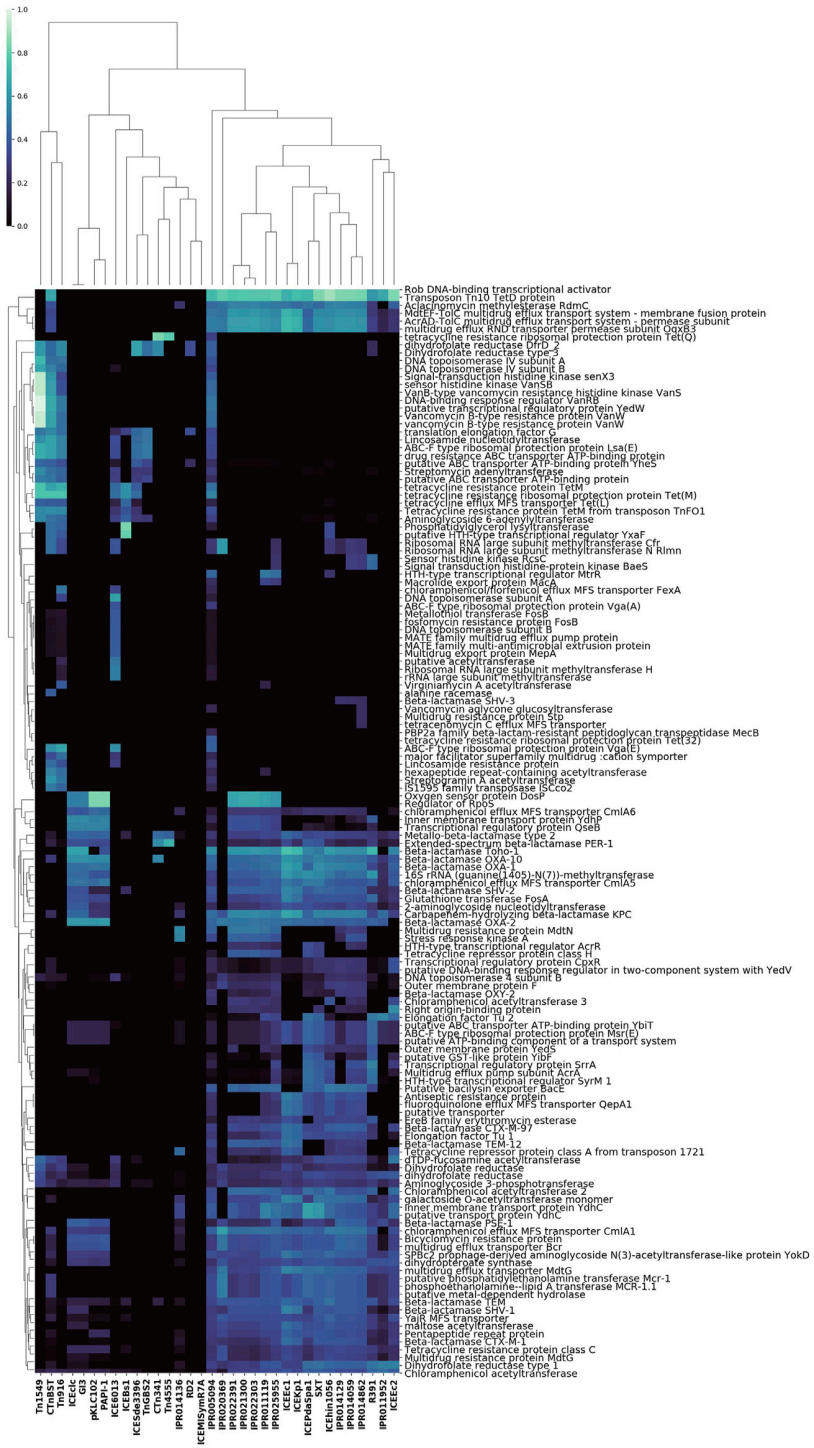
Co-occurrence of AMR proteins with conjugative features, for the 138 AMR proteins observed in conjugative genomes with a frequency greater than 5x the frequency of observation in non-conjugative genomes. Of these AMR proteins, 43 are *only* observed in conjugative genomes.

**FIGURE 5:**
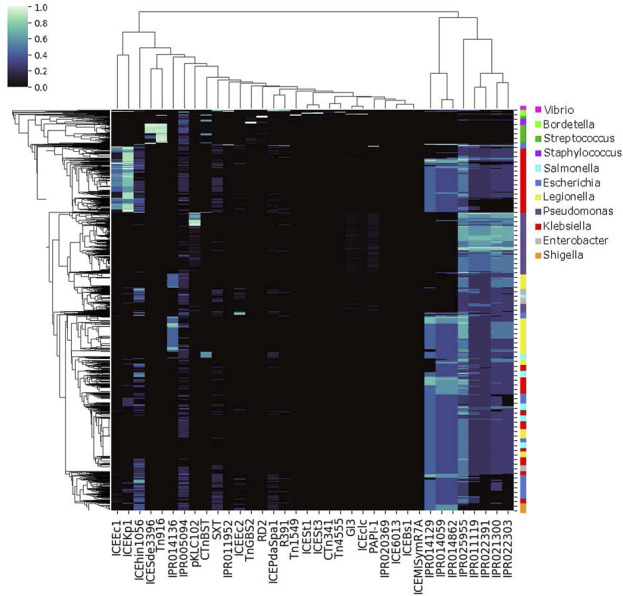
Genome-IPR Co-Occurence Map. Co-occurrence of conjugation features by genome, for the 6,000 genomes with the highest cargo protein fraction and the 500 genomes with the rarest conjugation features (see text). Co-occurrence of particular conjugation features does vary by taxonomic group.

Conjugative genomes were tabulated by genus as shown in Table 2. Observed conjugative features from Table 1 were tabulated by genome for the analysis shown in Figures 1, 5. Similarly, proteins assigned AMR-associated annotations were tabulated by number of conjugative and non-conjugative genomes, and the fraction of unique sequences observed in plasmids was tabulated as well (See Figure 4).

#### 4.3.6 Hierarchical clustering and co-occurrence

Hierarchical clustering was used to characterize the co-occurrence of proteins or genomes by conjugation feature (Figures 1, 4, 5. The co-occurrence matrix was generated using the Seaborn clustermap algorithm, which performs single linkage clustering to generate heatmaps and dendrograms (Waskom, 2015). The vector of features used to generate the heatmap shown in Figure 2 was the vector of conjugation features for each genome, which in order to compute the (Euclidean) distance between each genome. Again, Seaborn clustermap was used for hierarchical clustering and visualization.

## Data Availability

The original contributions presented in the study are included in the article Supplementary Material and at https://github.com/Ruiz-HCI-Lab/ggMOB. Further inquiries can be directed to the corresponding author.

## Supplementary material

Supplementary Figure S1 Co-occurrence of AMR proteins with conjugative features.Co-occurrence of AMR proteins with conjugative features for the 29 AMR proteins observed in non-conjugative genomes with a frequency at least 5x the frequency of observation in conjugative genomes..

Supplementary Figure S2 Heatmap of Genome-Genome Distances Based on Conjugation Feature Profiles. Genome-genome Euclidean distance between vectors of conjugation features for the 6500 genomes used in 5. For visualization purposes, 1 in 75 labels were rendered on each axis. The figure shows that different sets of genomes cluster based on different co-occurring conjugation features..
